# Needle tract seeding after fine-needle aspiration for pancreatic cancer assessed by endoscopy and endoscopic ultrasound

**DOI:** 10.1055/a-2738-7245

**Published:** 2025-11-19

**Authors:** Koichi Soga, Fuki Hayakawa, Ikuhiro Kobori, Hidehiro Tajima, Shinichi Ban, Hideyuki Yoshitomi, Masaya Tamano

**Affiliations:** 1 26263 Department of Gastroenterology, Dokkyo Medical University Saitama Medical Center, Koshigaya, Japan; 2 26263 Department of Surgery, Dokkyo Medical University Saitama Medical Center, Koshigaya, Japan; 3 Department of Pathology, Dokkyo Medical University Saitama Medical Center, Koshigaya, Japan

Needle tract seeding (NTS) is a rare but potentially serious complication of endoscopic ultrasound-guided fine-needle aspiration (EUS-FNA) for pancreatic ductal adenocarcinoma (PDAC), particularly in cases involving transgastric puncture 1 . This case demonstrates that NTS can develop years after EUS-FNA and that detailed endoscopic and endoscopic ultrasound (EUS) evaluation can guide curative resection through conversion surgery.

A patient in their 50s underwent curative distal pancreatectomy for PDAC, which had been histologically diagnosed using transgastric EUS-FNA ( Fig. 1 ). Two and a half years later, imaging revealed new neoplastic lesions in the stomach and liver ( Fig. 2 ). Positron emission tomography–computed tomography (PET–CT) revealed an elevated standardized uptake value (SUV), suggesting NTS and liver metastasis. After 22 months of chemotherapy for the metastasis, the SUV decreased significantly, allowing for conversion surgery ( Fig. 3 ). Before conversion surgery, esophagogastroduodenoscopy confirmed a submucosal tumor (SMT)-like lesion corresponding to the puncture site. EUS revealed a 15-mm irregular hypoechoic mass localized from the muscularis propria to the serosa, without mucosal invasion. Clips and tattoo marks were placed on the SMT-like lesion in the stomach for localization ( Fig. 4 and Video 1 ). After surgery, the macroscopic pathological specimen revealed a solid greyish-white tumor, which corresponded well with the EUS findings. Histological analysis confirmed moderately differentiated adenocarcinoma resembling the original PDAC. The lesion was confined to the muscularis propria and serosa, with negative resection margins ( Fig. 5 ).

**Fig. 1:**
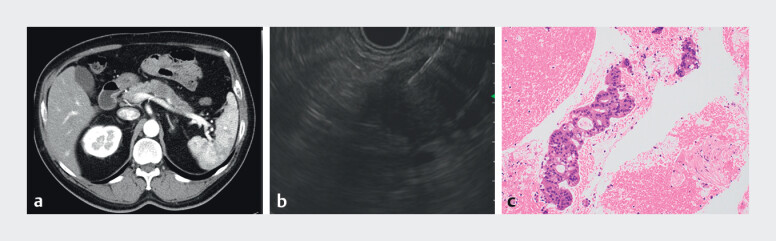
Images of pancreatic body cancer prior to the initial surgery: Representative imaging findings. **a** Contrast-enhanced computed tomography (CECT) demonstrating a hypovascular mass in the pancreatic body. Pancreatic body tumors exhibit poor contrast enhancement. **b** Endoscopic ultrasonography-guided fine-needle aspiration (EUS-FNA) image of the pancreatic body mass. A hypoechoic lesion with irregular margins was clearly visualized and targeted using EUS guidance. EUS-FNA was performed via the transgastric approach using a 22-gauge needle, with three passes for tissue acquisition. **c** Histopathological findings of the pancreatic tumor obtained using EUS-FNA at initial diagnosis. The specimen revealed clusters of atypical epithelial cells forming glandular structures with enlarged hyperchromatic nuclei and irregular nuclear contours consistent with adenocarcinoma. Haematoxylin and eosin staining was performed (×200).

**Fig. 2:**
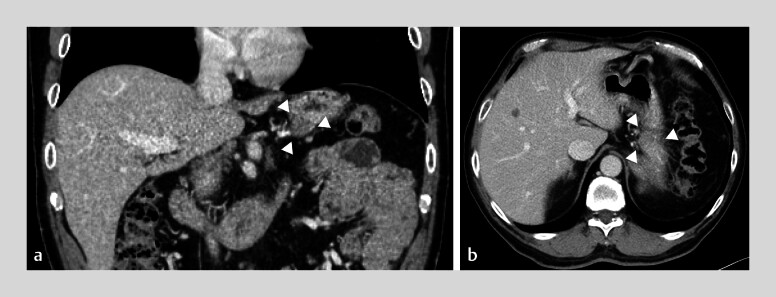
Needle tract seeding (NTS) along the endoscopic ultrasonography-guided fine-needle aspiration (EUS-FNA) puncture route: Imaging findings 2.5 years after initial EUS-FNA for pancreatic body adenocarcinoma (PDAC). **a** Coronal and **b** axial contrast-enhanced computed tomography (CECT) showing a tissue lesion along the gastric wall (arrowheads), consistent with NTS, following initial EUS-FNA for PDAC.

**Fig. 3:**
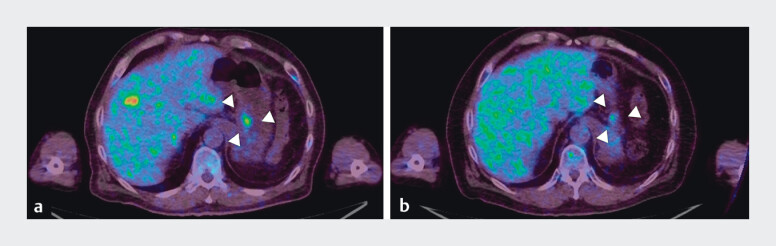
Positron emission tomography–computed tomography (PET–CT) (delayed-phase) demonstrating increased fluorodeoxyglucose (FDG) uptake at the site of needle tract seeding (NTS) **a** 2.5 and **b** 4.5 after initial endoscopic ultrasonography-guided fine-needle aspiration (EUS-FNA) for pancreatic body adenocarcinoma (PDAC). **a** PET-CT image obtained 2.5 years after initial EUS-FNA for PDCA. Increased FDG uptake was observed along the gastric wall, corresponding to the NTS identified on CT. **b** Compared to prior PET-CT findings, a marked reduction in FDG uptake was observed at the seeding site along the gastric wall, indicating a favorable metabolic response to chemotherapy.

**Fig. 4:**
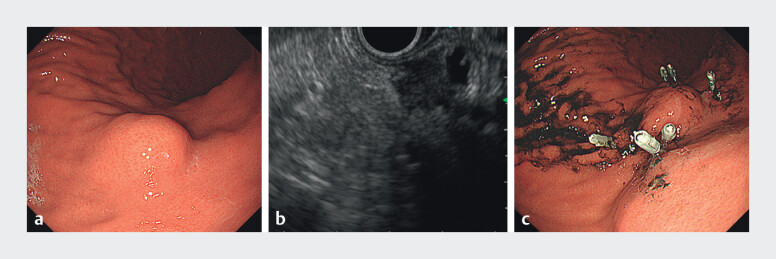
Preoperative endoscopic **a** and **c** and endoscopic ultrasonography (EUS) **b** assessment of needle tract seeding (NTS) following chemotherapy: submucosal tumor (SMT)-like lesions with extramural infiltration were evident on EUS. **a** Esophagogastroduodenoscopy performed before conversion surgery for NTS. An SMT-like lesion was observed on the posterior gastric wall at the prior transgastric puncture site without evidence of mucosal ulceration or disruption. **b** EUS image obtained during the same session. The lesion appeared to originate from the fourth layer (muscularis propria) of the gastric wall and demonstrated clear extramural extension beyond the gastric serosa. **c** Clip and tattoo marks were placed on the stomach to localize the SMT-like lesion.

Needle tract seeding after fine-needle aspiration for pancreatic cancer assessed by endoscopy and endoscopic ultrasound.Video 1

**Fig. 5:**
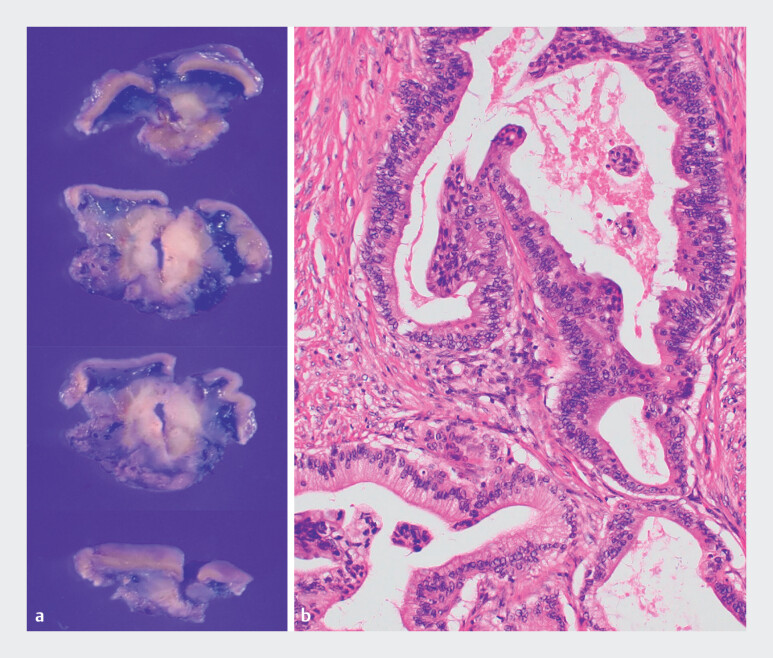
Surgical resection of needle tract seeding (NTS) after endoscopic ultrasonography-guided fine-needle aspiration (EUS-FNA): intraoperative clip-guided local resection and pathological confirmation. **a** Macroscopic view of the resected gastric specimen. Resection was guided by the preoperative placement of marking clips under EUS guidance, allowing for accurate and complete excision of the targeted lesion. The cut surface showed a whitish, well-demarcated tumor, consistent with grossly complete resection. A central notch was made in the specimen to facilitate the pathological assessment. **b** Histopathological findings from the resected specimen (hematoxylin and eosin staining, ×100). The tumor exhibited well-formed glandular structures that were morphologically identical to those observed in previously resected pancreatic ductal adenocarcinomas, confirming the diagnosis of NTS originating from pancreatic cancer.

EUS-FNA is a well-established diagnostic modality for PDAC, offering high sensitivity and specificity. Several studies have reported a non-negligible incidence of NTS, ranging from 0.3% to 3.8% 1 2 3 . EUS plays a pivotal role in layer-specific localization, supporting the hypothesis that tumor cell implantation occurs in the muscularis propria and expands outward. Preoperative EUS imaging and endoscopic markings facilitated precise resection. This case highlights the clinical value of EUS, not only for diagnostic purposes but also for surgical planning in NTS.

Endoscopy_UCTN_Code_CPL_1AL_2AD

## Notes

Endoscopy E-Videos https://eref.thieme.de/e-videos
*E-Videos* is an open access online section of the journal *Endoscopy* , reporting on interesting cases and new techniques in gastroenterological endoscopy. All papers include a high-quality video and are published with a Creative Commons CC-BY license. Endoscopy E-Videos qualify for HINARI discounts and waivers and eligibility is automatically checked during the submission process. We grant 100% waivers to articles whose corresponding authors are based in Group A countries and 50% waivers to those who are based in Group B countries as classified by Research4Life (see: https://www.research4life.org/access/eligibility/ ). This section has its own submission website at https://mc.manuscriptcentral.com/e-videos .
